# Peroxiredoxin 2 activates microglia by interacting with Toll-like receptor 4 after subarachnoid hemorrhage

**DOI:** 10.1186/s12974-018-1118-4

**Published:** 2018-03-19

**Authors:** Yue Lu, Xiang-Sheng Zhang, Zi-Huan Zhang, Xiao-Ming Zhou, Yong-Yue Gao, Guang-Jie Liu, Han Wang, Ling-Yun Wu, Wei Li, Chun-Hua Hang

**Affiliations:** 1 Department of Neurosurgery, Drum Tower Hospital, Medical School of Nanjing University, Nanjing, China; 2 Department of Neurosurgery, Zhongdu Hospital, Bengbu, China; 3 Department of Neurosurgery, Changzheng Hospital, Second Military Medical University, Shanghai, China; 4 Department of Neurosurgery, Jinling Hospital, School of Medicine, South Medical University, Nanjing, China

**Keywords:** Subarachnoid hemorrhage, Peroxiredoxin 2, Microglial activation, Toll-like receptor 4, Neuronal apoptosis

## Abstract

**Background:**

Peroxiredoxin (Prx) protein family have been reported as important damage-associated molecular patterns (DAMPs) in ischemic stroke. Since peroxiredoxin 2 (Prx2) is the third most abundant protein in erythrocytes and the second most protein in the cerebrospinal fluid in traumatic brain injury and subarachnoid hemorrhage (SAH) patients, we assessed the role of extracellular Prx2 in the context of SAH.

**Methods:**

We introduced a co-culture system of primary neurons and microglia. Prx2 was added to culture medium with oxyhemoglobin (OxyHb) to mimic SAH in vitro. Neuronal cell viability was assessed by lactate dehydrogenase (LDH) assay, and neuronal apoptosis was determined by TUNEL staining. Inflammatory factors in culture medium were measured by ELISA, and their mRNA levels in microglia were determined by qPCR. Toll-like receptor 4 knockout (TLR4-KO) mice were used to provide TLR4-KO microglia; ST-2825 was used to inhibit MyD88, and pyrrolidine dithiocarbamate (PDTC) was used to inhibit NF-κB. Related cellular signals were analyzed by Western blot. Furthermore, we detected the level of Prx2 in aneurysmal SAH patients’ cerebrospinal fluids (CSF) and compared its relationship with Hunt-Hess grades.

**Results:**

Prx2 interacted with TLR4 on microglia after SAH and then activated microglia through TLR4/MyD88/NF-κB signaling pathway. Pro-inflammatory factors were expressed and released, eventually caused neuronal apoptosis. The levels of Prx2 in SAH patients positively correlated with Hunt-Hess grades.

**Conclusions:**

Extracellular Prx2 in CSF after SAH is a DAMP which resulted in microglial activation via TLR4/MyD88/NF-κB pathway and then neuronal apoptosis. Prx2 in patients’ CSF may be a potential indicator of brain injury and prognosis.

**Electronic supplementary material:**

The online version of this article (10.1186/s12974-018-1118-4) contains supplementary material, which is available to authorized users.

## Background

Emerging studies have shown that peroxiredoxin family plays a dual role in many diseases [1–4]. The intracellular Prxs are hydrogen peroxide and organic hydroperoxide scavengers, which exert protective effects on oxidative stress. However, Prxs, especially Prx5/6, are proven to be strong damage-associated molecular patterns (DAMPs) in ischemic stroke [5]. Prx1-mediated activation of TLR4/NF-κB contributes to neuroinflammatory responses in intracerebral hemorrhage [6]. Recent study has demonstrated that Prx2 was the second most protein in the cerebrospinal fluid (CSF) of traumatic brain injury and SAH patients [7]. In Prxs family, Prx2 was the third abundant protein in erythrocytes and it was also highly expressed in neurons [8, 9]. So it is obvious that the lytic red blood cells and damaged neurons will release a great amount of Prx2 into the subarachnoid space after SAH. However, the role of extracellular Prx2 after SAH has not been clarified.

In this study, we investigated whether the extracellular Prx2 has an effect on neuronal apoptosis after SAH. A primary neuron and microglia co-culture model was introduced to mimic SAH in vitro. Since microglia is an important initiator cell in neuroinflammation, we focused on the interaction between Prx2 and microglial activation.

## Methods

### Animals

All animal procedures were approved by the Ethics Review Committee for Animal Experimentation at Drum Tower Hospital (Nanjing, China) and performed in accordance with the institutional guidelines. Neonatal C57BL/6JNju mice were sacrificed for primary cortical neurons and microglia. For primary TLR4-KO microglia, neonatal C57BL/10ScNJNju mice were used. Two strains of mice were purchased from Nanjing Biomedical Research Institute of Nanjing University.

### Primary cell culture

Primary cortical neural cells were cultured as described previously [10]. In brief, cerebral cortex was isolated from brains of neonatal (1–3 days) mice. The leptomeninges and white matter were removed, and brain tissue were digested with 0.125% trypsin (Gibco) for 5 min at 37 °C. Subsequently, the suspension was filtered through a 40-μm strainer (Millipore) and centrifuged at 1500 r/min for 10 min. The remaining cells were resuspended in Dulbecco’s modified Eagle’s medium (DMEM) with 10% fetal bovine serum (FBS) and penicillin-streptomycin. For neurons, cell suspensions were seeded into poly-d-lysine-coated six-well plates, and their culture medium was replaced after 2 h with Neurobasal Medium containing 0.5 mmol/L GlutaMAX-I and 2% B27 supplement (Gibco). Neuronal cultures were used on day 8 in vitro (DIV8). Primary microglia were obtained as described [11]; cells were seeded in flasks coated with poly-d-lysine to obtain mixed glial cultures. When the glial cultures reached confluency for 3 days, the flasks were shaken 2 h at 250 rpm. The floating cells were collected and seeded in six-well plates to obtain microglia. Microglial cultures were used on DIV14. For neurons and microglia co-culture system, microglia were seeded in Transwell (Corning, pore size = 0.4 μm) upper chamber and the neurons were seeded in the plates. Co-culture medium was DMEM with 10% FBS. The serum was reduced to 2% before any treatment. The co-culture system was harvested 24 h after indicated intervention. The purity of primary neuron and microglia was more than 90% (Additional file 1: Figure S1).

### Preparation of oxyhemoglobin

Mouse hemoglobin (Sigma) was used to produce oxyhemoglobin as per the manufacturer’s instruction. In brief, the reduced hemoglobin was prepared by gel filtrating 1 mmol/L hemoglobin solution with sodium dithionite in a column containing Sephadex G-25 (Sigma). Then, the reduced hemoglobin was saturated with oxygen gas. Sodium dithionite was then removed by dialysis against 100 volumes of oxygen-saturated phosphate buffer. Oxyhemoglobin were achieved and stored at − 80 °C.

### Oxyhemoglobin-incubated in vitro SAH model and drug administration

To mimic SAH in vitro, the co-culture system was exposed to oxyhemoglobin (OxyHb) at a concentration of 25 μmol/L. The treatment groups were prepared with OxyHb-exposed neuron-microglia co-culture systems respectively adding 1, 5, and 10 μg/mL recombinant Prx2 (Abcam) for 24 h. MyD88 inhibitor ST-2825 (MCE) was premixed with culture medium at a concentration of 10 and 100 μmol/L, and the NF-κB inhibitor pyrrolidine dithiocarbamate (PDTC) was premixed with microglial culture medium at a concentration of 80 μmol/L 24 h before the in vitro SAH model was induced respectively.

### Cell viability assay

The Cytotoxicity Detection Kit (LDH) (Roche) was used to measure neural cell viability as per the manufacturer’s instruction. After treatment, 100 μL of culture medium was transferred to a 96-well plate; the reaction mixture was added to each well and incubated for 30 min at room temperature. The absorbance was measured at a wavelength of 490 nm, and the reference wavelength was 690 nm.

### Real-time polymerase chain reaction

RNA extracted using TRIzol Reagent (TAKARA) was reverse transcribed into cDNA with the Reverse Transcriptase Reagent (TAKARA). Quantitative real-time PCR analysis was performed with UltraSYBR Mixture using the LightCycler 96 Real-Time PCR System (Roche). The primers are as follows: IL-1β, 5′-GCCTGTGTTTTCCTCCTTGC-3′ (forward), 5-TGCTGCCTAATGTCCCCTTG-3′ (reverse); TNF-α, 5′-CGGGCAGGTCTACTTTGGAG-3′ (forward), 5′-ACCCTGAGCCATAATCCCCT-3′ (reverse); IL-6, 5′-GAGACTTCCATCCAGTTGCCT-3′ (forward), 5′-TGGGAGTGGTATCCTCTGTGA-3′ (reverse); and Rpl5, 5′-GGAAGCACATCATGGGTCAGA-3′ (forward), 5′-TACGCATCTTCATCTTCCTCCATT-3′ (reverse). Rpl5 was used as housekeeping gene. After 95 °C for 30 s, 40 PCR cycles were performed, each consisting of a denaturation step (95 °C, 5 s) and an annealing step (60 °C, 30 s).

### Enzyme-linked immunosorbent assay

The primary microglial culture medium of each group was collected, and the quantities of IL-1β, TNF-α, and IL-6 were determined using enzyme-linked immunosorbent assay kits (Boster) according to the manufacturer’s instructions.

### Immunofluorescence staining

Immunofluorescence staining was performed according to our previous study. Briefly, cultured cells on coverslips were fixed in 4% paraformaldehyde. Following treatment with 0.1% Triton X-100, the samples were blocked by 5% bovine serum albumin (BSA) prior to incubation with primary antibody overnight. The samples were washed three times with 0.5% phosphate-buffered saline with Tween-20 (PBST) and then were incubated with proper secondary antibodies. After three washes again, the coverslips were counterstained by 4,6-diamidino-2-phenylindole (DAPI) for 2 min. The following antibodies were used: anti-Iba1 (1:200, Abcam) and NeuN (1:200, Millipore). Pictures were acquired with a fluorescence microscope (Zeiss) under the same exposure time 0.5 s.

### TUNEL staining

Terminal deoxynucleotidyl transferase-mediated dUTP nick-end labelling (TUNEL) staining was conducted by using a TUNEL detection kit according to the manufacturer’s instructions (Roche). Coverslips were incubated with primary antibody against NeuN (1:100, Millipore) at 4 °C overnight. After washed three times with PBST, the coverslips were incubated with TUNEL reaction mixture for 45 min prior to be counterstained by DAPI. The positive cells were identified, counted, and analyzed by two investigators blinded to the grouping.

### Western blot analysis

Cells after indicated treatment were washed three times with PBS and lysed in radioimmunoprecipitation assay buffer (RIPA) containing protease and phosphatase inhibitor cocktails (Roche). Protein concentrations were determined with the BCA kit (Beyotime). Equal amounts of protein (10 μg) per lane were separated by SDS-polyacrylamide gel and transferred to a polyvinylidene difluoride (PVDF) membrane. The membrane was blocked in 5% skim milk for 2 h at room temperature and incubated overnight at 4 °C with primary antibodies against Iba-1 (1:1000, Abcam), MyD88 (1:2000, Abcam), p65 (1:1000, Cell Signaling Technology), Prx2 (1:5000, Abcam), and TLR4 (1:200, Santa Cruz) in 0.1% TBST containing 5% BSA. After the membrane was washed, it was incubated with HRP-conjugated secondary antibody for 2 h at room temperature. Detection was performed by Immobilon Western Chemiluminescent HRP Substrate (Millipore), according to the manufacturer’s instruction. Band intensities were quantified using the ImageJ software.

### Patient recruitment and CSF collection

Eight control patients and 24 SAH patients from Drum Tower Hospital were included prospectively after written informed consent was obtained from all patients or their family members. This study was conducted in accordance with the Declaration of Helsinki and was approved by the Ethics Committee of Drum Tower Hospital. CSF samples were collected through lumbar puncture between 24 and 72 h after the SAH. Immediately after collection, samples were centrifuged at 3000 rpm for 10 min at 4 °C and the supernatants were collected. An equal amount (50 μL) of sample per lane was added and detected by Western blot.

### Statistical analysis

All data were expressed as the mean ± SD. Statistical comparisons were performed using one-way ANOVA. Differences between experimental groups were determined by Student’s *t* test. A value of *P* < 0.05 was considered statistically significant.

## Results

### Prx2 aggravated neuronal damage in neuron-microglia co-culture system after SAH

**Fig. 1:**
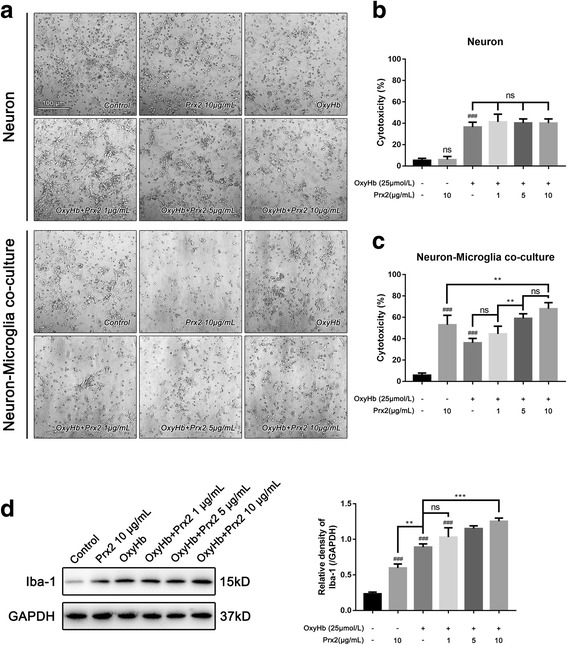
Prx2-induced neuronal cytotoxicity was mediated by microglia. **a** The phase-contrast photomicrographs of cultured neuron, scale bar = 100 μm. Treated by Prx2, cultured neuron did not show overt neuronal damage. In neuron-microglia co-culture system, the Prx2 exerted neurotoxicity. **b** Prx2 had no neurotoxicity when treated with cultured neurons after SAH. **c** Co-cultured with microglia, the neurons were injured by Prx2 in a dose-dependent manner. **d** The microglia were activated by Prx2 after SAH. ^###^*P* < 0.001 vs control; **P < 0.01, ***P < 0.001, ^ns^*P* > 0.05 vs indicated groups. Bars represent the mean ± SD. *N* = 6 in each group

Single-neuron culture and neuron-microglia co-culture system were treated with different doses of recombinant Prx2 respectively. The neuronal damage was aggravated after incubation with oxyhemoglobin (OxyHb) for 24 h, and the damage became more severe when it was co-cultured with microglia (Fig. 1a). In neuron-microglia co-culture system, exposure to Prx2 10 μg/mL alone could cause neuronal disintegration. As shown in Fig. 1b, treating single-neuron culture with Prx2 alone did not affect the neuronal cytotoxicity. Incubation with OxyHb produced obvious neuronal cytotoxicity; however, when compared with the OxyHb group, there were no significant differences in the OxyHb+Prx2 groups. In neuron-microglia co-culture system, a Prx2 dose-dependent neurotoxicity was observed. The OxyHb+Prx2 10 μg/mL group was the most severe one. Interestingly, treating the co-culture system with Prx2 alone could also cause neurotoxicity (Fig. 1c). To determine the microglial activation, we assessed the Iba-1 expression in primary microglia by Western blot. As shown in Fig. 1d, Prx2 significantly increased the expression of Iba-1 after in vitro SAH.

### Prx2 facilitated the synthesis and secretion of pro-inflammatory cytokines by microglia after SAH in vitro

**Fig. 2:**
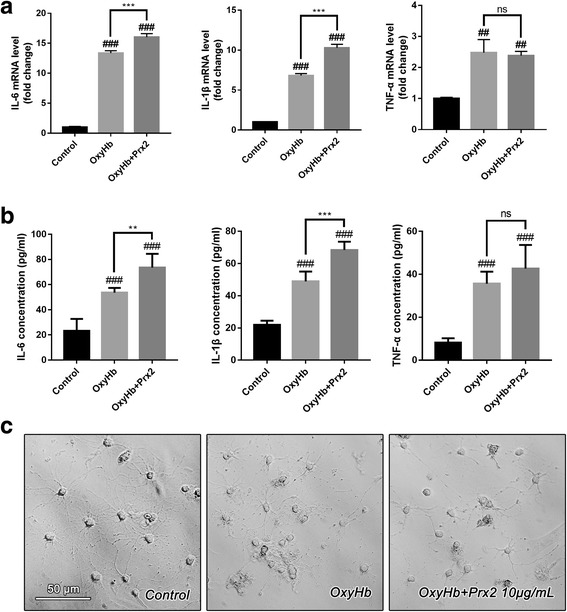
The microglia were activated by Prx2 and polarized to M1-like microglia. **a** The mRNA level of IL-1β, IL-6, and TNF-α in microglia increased dramatically after SAH. The IL-1β and IL-6 mRNA level in OxyHb + Prx2 10 μg/mL group increased significantly after Prx2 treatment when compared with OxyHb group. The mRNA level of TNF-α remained unchanged. **b** The concentrations of IL-1β, IL-6, and TNF-α in culture medium of microglia were determined by ELISA. Consistent with the mRNA level, the IL-1β and IL-6 concentrations in OxyHb + Prx2 10 μg/mL group increased significantly after Prx2 treatment compared with the OxyHb group. However, the concentration of TNF-α remained unchanged. **c** The phase-contrast photomicrographs of cultured microglia. Ramified microglia in the control group became less branching after SAH. Incubated with Prx2, the number of microglial branches became more less. Scale bar = 50 μm. ^##^*P* < 0.01, ^###^*P* < 0.001 vs control; **P < 0.01, ***P < 0.001, ^ns^*P* > 0.05 vs indicated groups. Bars represent the mean ± SD. *N* = 6 in each group

To investigate the mechanism of microglia-dependent Prx2-induced neurotoxicity, we detect the interleukin 1β (IL-1β), interleukin 6 (IL-6), and tumor necrosis factor-α (TNF-α) mRNA expression in microglia and their concentrations in the co-culture medium. As shown in Fig. 2a, the IL-1β, IL-6, and TNF-α mRNA level in cultured microglia were elevated after OxyHb incubation for 24 h. Moreover, incubation of OxyHb along with 10 μg/mL Prx2 promoted the mRNA expression of IL-1β and IL-6. However, TNF-α mRNA level remained unchanged in the OxyHb+Prx2 group compared with the OxyHb group. Consistent with the results above, the concentration of IL-1β and IL-6 in the microglial culture medium were significantly increased after SAH. Prx2 treatment markedly increased their concentration in culture medium when compared with the OxyHb group (Fig. 2b). Phase-contrast microscope images revealed the morphological remodeling of microglia in response to OxyHb and OxyHb+Prx2 treatment. In the control group, microglia have highly ramified morphology with thin processes. Upon OxyHb stimuli, the processes of microglia retracted and thickened, which indicated the microglial activation and acquirement of the ability of secreting pro-inflammatory cytokines. Compared with the OxyHb group, incubation of OxyHb+Prx2 further activated the microglia (Fig. 2c).

### TLR4/MyD88/NF-κB signaling pathway was involved in the Prx2-induced microglial activation after SAH in vitro

**Fig. 3:**
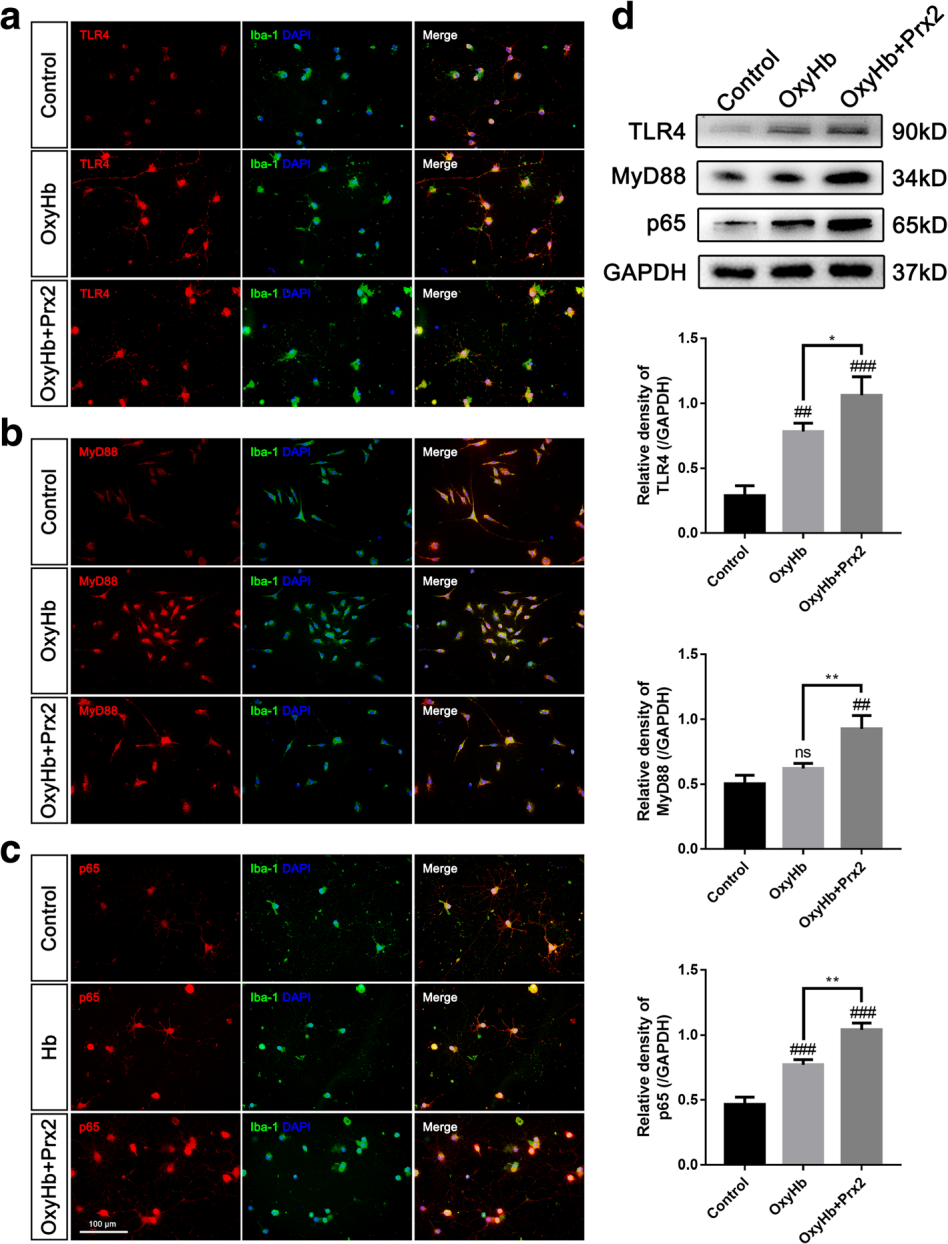
Prx2 increased the expression of TLR4, MyD88, and p65 in microglia. **a–c** Immunofluorescence staining showed that the expression of TLR4, MyD88, and p65 increased in the OxyHb group and OxyHb + Prx2 10 μg/mL group. Scale bar = 100 μm. **d** Western blot showing that Prx2 further increased the expression of TLR4, MyD88, and p65 after SAH compared with OxyHb group. ^##^*P* < 0.01, ^###^*P* < 0.001, ^ns^*P* > 0.05 vs control; *P < 0.05, **P < 0.01 vs indicated groups. Bars represent the mean ± SD. *N* = 6 in each group

**Fig. 4:**
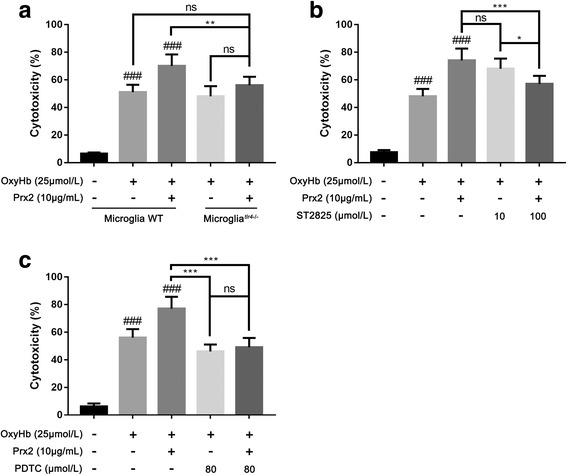
Prx2-induced neurotoxicity was eliminated by microglial *Tlr4* knockout or inhibition of MyD88. **a** When co-cultured with TLR4-KO microglia, the neurotoxicity caused by Prx2 was eliminated after SAH. **b** Inhibition of MyD88 in microglia by ST-2825 in 100 μmol/L eliminated the Prx2-induced microglia-mediated neurotoxicity. **c** Inhibition of NF-κB by PDTC in 80 μmol/L significantly remitted the neuronal cytotoxicity caused by Prx2. ^###^*P* < 0.001 vs control; *P < 0.05, **P < 0.01, ***P < 0.001, and ^ns^*P* > 0.05 vs indicated groups. Bars represent the mean ± SD. *N* = 6 in each group

The TLR4/MyD88/NF-κB signaling pathway was involved in the activation of microglia. Since peroxiredoxin 1 (Prx1) could interact with TLR4, we assumed that Prx2 may activate microglia after SAH by interacting with TLR4. We detected the expression of TLR4, MyD88, and p65 in cultured microglia by immunofluorescence and Western blot. After SAH, the expression of TLR4, MyD88, and p65 were elevated (Fig. 3a). The Western blot results showed that Prx2 increased the expression of TLR4, MyD88, and p65 when compared with the OxyHb group (Fig. 3b). To confirm the effects, we co-cultured neuron with TLR4-KO microglia and ST-2825-treated microglia. We found that TLR4 KO eliminated the Prx2-induced neuronal cytotoxicity (Fig. 4a). Inhibition of MyD88 by ST-2825 at the concentration of 100 μmol/L significantly remitted the neuronal cytotoxicity caused by Prx2 (Fig. 4b). PDTC was used to inhibit NF-κB activity in microglia. After treated by PDTC at the concentration of 80 μmol/L, the Prx2-induced microglia-mediated neuronal cytotoxicity was eliminated (Fig. 4c).

### TLR4 knockout microglia lose the ability to induce neuronal apoptosis caused by Prx2

**Fig. 5:**
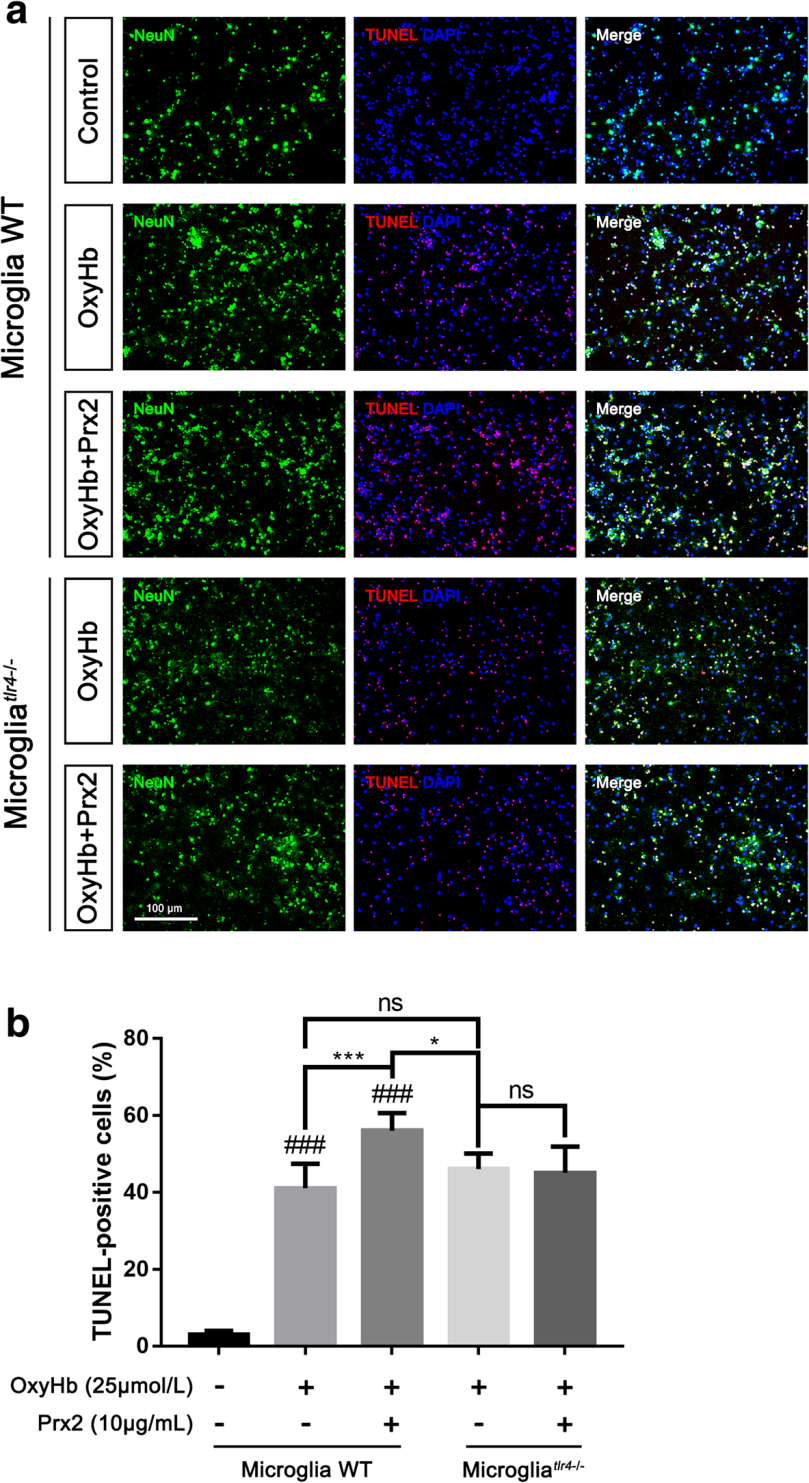
TLR4-KO in microglia attenuated Prx2-induced microglia-mediated neuronal apoptosis. **a** TUNEL-staining showed that the number of apoptotic neurons decreased when co-cultured with TLR4-KO microglia after Prx2 exposure. **b** Quantitative analysis of the proportion of apoptotic neurons. ^###^*P* < 0.001 vs control; *P < 0.05, ***P < 0.001, and ^ns^*P* > 0.05 vs indicated groups. Bars represent the mean ± SD. *N* = 6 in each group

TUNEL staining was performed to demonstrate the role of Prx2-induced microglia-mediated neuronal injury (Fig. 5a). In neuron-microglia co-culture system, the fraction of apoptotic neurons was significantly increased after in vitro SAH model was induced. Compared with the OxyHb group, the number of apoptotic neurons was increased in the OxyHb+Prx2 group. We then co-cultured neurons with TLR4-KO microglia; the Prx2-induced microglia-mediated neuronal apoptosis was attenuated (Fig. 5b).

### The level of Prx2 in cerebrospinal fluid of SAH patients correlated with Hunt-Hess grades

**Fig. 6:**
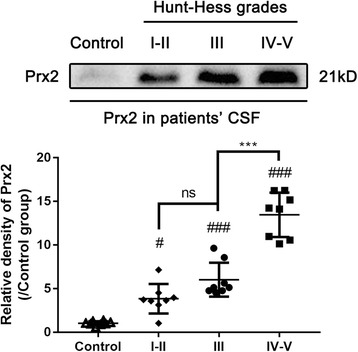
The Prx2 level in aneurysmal SAH patients’ CSF positively correlated with Hunt-Hess grades. The Prx2 level in patients’ CSF was determined by Western blot. Prx2 in the CSF of SAH patients increased significantly compared with the control group. In grade IV–V patients, the level of Prx2 was higher than grade I–III patients. ^#^*P* < 0.05, ^###^*P* < 0.001 vs control; ***P < 0.001 and ^ns^*P* > 0.05 vs indicated groups. Bars represent the mean ± SD. *N* = 6 in each group

To investigate the relationship between Prx2 in CSF with the severity of brain injury, we detected the Prx2 level in aneurysmal SAH patients’ CSF by Western blot. The CSF collected from patients who underwent hip arthroplasty before surgery were used as control. We found that the Prx2 level in CSF after SAH was elevated significantly compared with the control group. There were no obvious differences between Hunt-Hess grade I–II group and grade III group. However, in grade IV–V group, the Prx2 level in CSF was increased significantly compared with grade III group (Fig. 6). These results indicated that the level of Prx2 in CSF of aneurysmal SAH patients was positively correlated with the severity of brain injury, and it may also correlate with prognosis of SAH patients.

## Discussion

Previous study has revealed that Prx2 is the second most protein in the CSF of traumatic brain injury and SAH patients [7]. It is not surprising that Prx2 concentration was high in CSF of SAH patients, because Prx2 is abundant in both erythrocytes and neurons [8, 9]. Although Prx2 is an organic hydrogen peroxide scavenger, it plays a role of damage-associated molecular pattern (DAMP) when it is released to the extracellular space. It has been proven that Prxs family proteins play important roles in ischemic stroke [5], and they function as DAMPs especially Prx5/6. In Prxs family, Prx1 and Prx2 share the most similarities on molecular structure [12]. Prx1 could increase the expression of TLR4 and activate TLR4/NF-κB signaling pathway [6]. In the context of SAH, Prx2 concentration in CSF correlates with patients’ Hunt-Hess grades, making it a potential indicator to judge the extent of brain injury and predictor of prognosis. We speculated that the level of Prx2 in CSF would positively correlate with the amount of bleeding and the number of necrotic neuron.

Though the polarization of microglia is controversial, the dichotomy between M1 and M2 phenotypes remains useful for explaining the function of microglia in brain diseases [13]. Prx2 promoted microglia synthesizing and secreting IL-1β and IL-6, but not TNF-α. Because IL-1β, IL-6, and TNF-α are the classical markers of M1-like microglia [14], Prx2 activated microglia to the M1-like phenotype. After oxidative stress and inflammatory insult, the signaling pathways leading to NF-κB and AP-1 activation are sometimes overlapping where both are involved in the induction and regulation of cytokines and chemokines. Although the TNF-α is known to be activated by NF-κB, their expression may be regulated in part by other independent pathways. Activator protein 1 (AP-1) is involved in LPS/TLR4-induced TNF and IL-6 production independent of NF-κB in primary human macrophages [15]. So it is possible that NF-κB signaling pathway was involved in Prx2-induced microglial activation but AP-1 was not.

In our co-culture system, neurons and microglia could not contact with each other physically. The microglia-mediated neurotoxicity must intermediate by some secreting substances. M1-like microglia has been recognized as a pro-inflammatory phenotype; the production of M1-like microglia such as reactive oxygen species (ROS), Fas-ligand (FasL), and nerve growth factor (NGF) can lead to neuronal apoptosis and necrosis [16, 17].

However, the crosstalk between the neurons and microglia is complicated; the in vitro model has limitations on interpretation of the comprehensive role of Prx2 after SAH. The inflammatory response after SAH involves the neurons, microglia, astrocytes, monocytes, etc. [18]. The high-mobility group protein B1 (HMGB1) released from necrotic neurons triggers the inflammatory response after SAH [19, 20]; however, Prxs in central nervous system should not be neglected. The pro-inflammatory effect of Prxs, especially Prx5/6, is stronger than HMGB1 in ischemic stroke [5]. TLR4 in microglia is a well-established membrane receptor that is able to activate microglia. The ligands to TLR4 contain protein, lipid, etc. [21]. Further studies are needed to decipher which is the most effective ligand that could activate microglia through TLR4 signaling pathway after SAH.

The involvement of different kinds of cell makes SAH a complicated pathophysiologic process. Our study focuses on the crosstalk between resident microglia and neurons in vitro. However, the monocytes and other immune cells were neglected in our study. The Ly6C^hi^ monocytes are able to translocate to brain tissue after inflammatory insults [17, 22]. They have the similar phenotype and function with the resident microglia. Prx2 may also interact with infiltrated monocytes and exert more powerful pro-inflammatory effects after SAH. On the other hand, the MAFB^hi^ monocytes have been proven that can phagocytose DAMPs including HMGB1, Prxs, and S100 through MSR1 to eliminate inflammation [23]. Because *Mafb* [24, 25] and *Msr1* [26] are highly expressed in microglia, the potential role of microglial phagocytosis is needed to be clarified.

## Conclusions

**Fig. 7:**
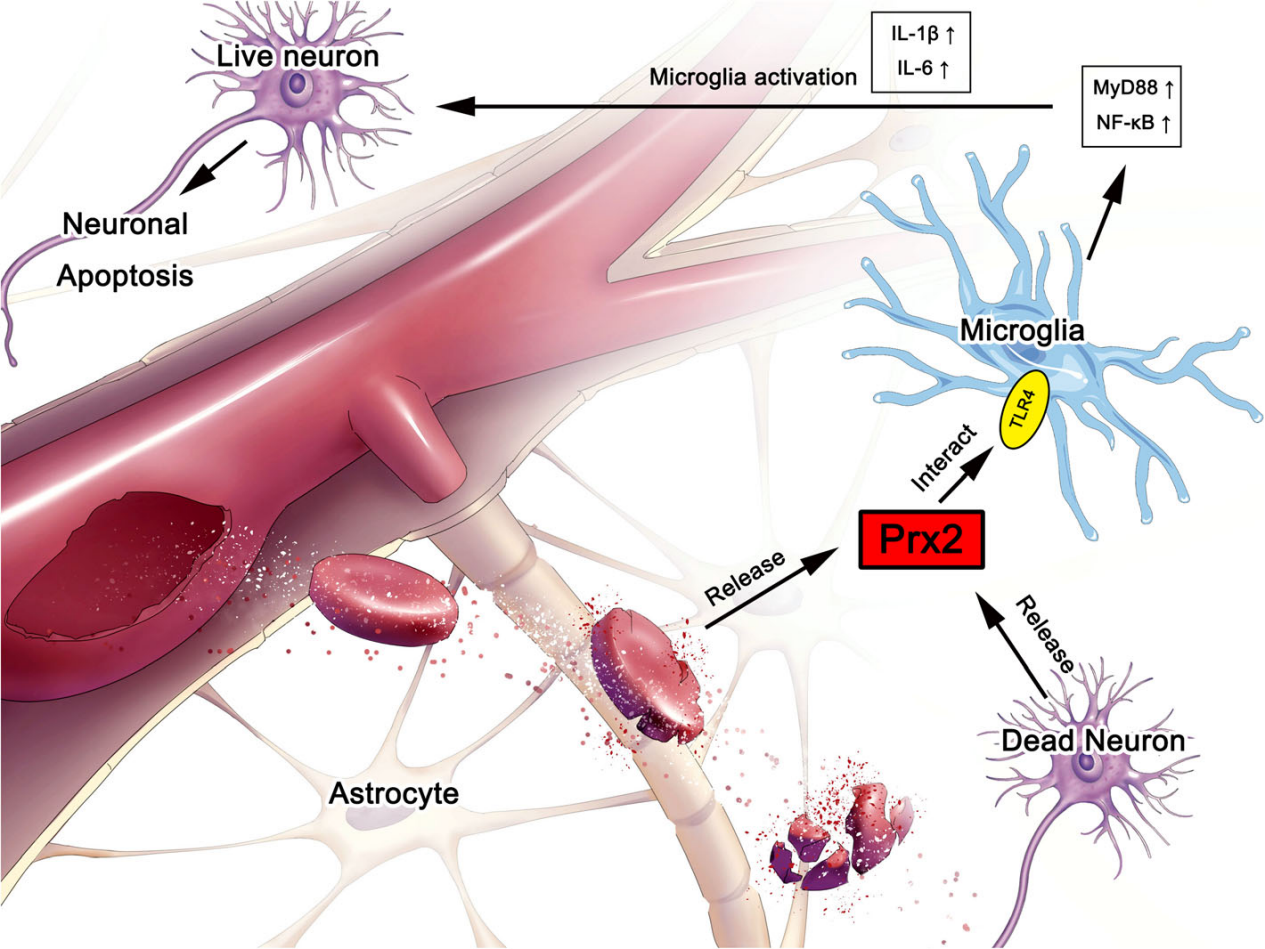
Prx2 activated microglia through TLR4/MyD88/NF-κB pathway and caused neuronal damage after SAH

Our research revealed that Prx2 released from lytic erythrocytes and damaged neurons after SAH activated microglia through TLR4/MyD88/NF-κB pathway. Then, the activated microglia elevated the expression of IL-1β and IL-6, which could result in neuronal apoptosis (Fig. 7). The level of Prx2 in SAH patients’ CSF positively correlated with Hunt-Hess grades.

## Additional file

Additional file 1: **Figure S1.** The purity of primary neuron and microglia. A–B. Immunofluorescence staining showed the neuron marker NeuN and microglia marker TMEM119 in primary cultured cells. The particle analysis was performed by ImageJ, and the purity of primary neuron and microglia was more than 90%. (DOCX 569 kb)

## Abbreviations

CSF: Cerebrospinal fluids
DAMP: Damage-associated molecular pattern
IL-1β: Interleukin 1β
IL-6: Interleukin 6
MyD88: Myeloid differentiation primary response 88
NF-κB: Nuclear factor-κB
Prx2: Peroxiredoxin 2
SAH: Subarachnoid hemorrhage
TLR4: Toll-like receptor 4
TNF-α: Tumor necrosis factor-α
