# Supplementary material for: Peroxiredoxin 2 activates microglia by interacting with Toll-like receptor 4 after subarachnoid hemorrhage
Source: J Neuroinflammation. 2018 Mar 19;15:87. doi: 10.1186/s12974-018-1118-4 (PMC5859544; doi:10.1186/s12974-018-1118-4)
Supplement: Supplementary file 1 — Figure S1. The purity of primary neuron and microglia. A–B. Immunofluorescence staining showed the neuron marker NeuN and microglia marker TMEM119 in primary cultured cells. The particle analysis was performed by ImageJ, and the purity of primary neuron and microglia was more than 90%. (DOCX 569 kb) [file 12974_2018_1118_MOESM1_ESM.docx]

**Supplemental Materials**


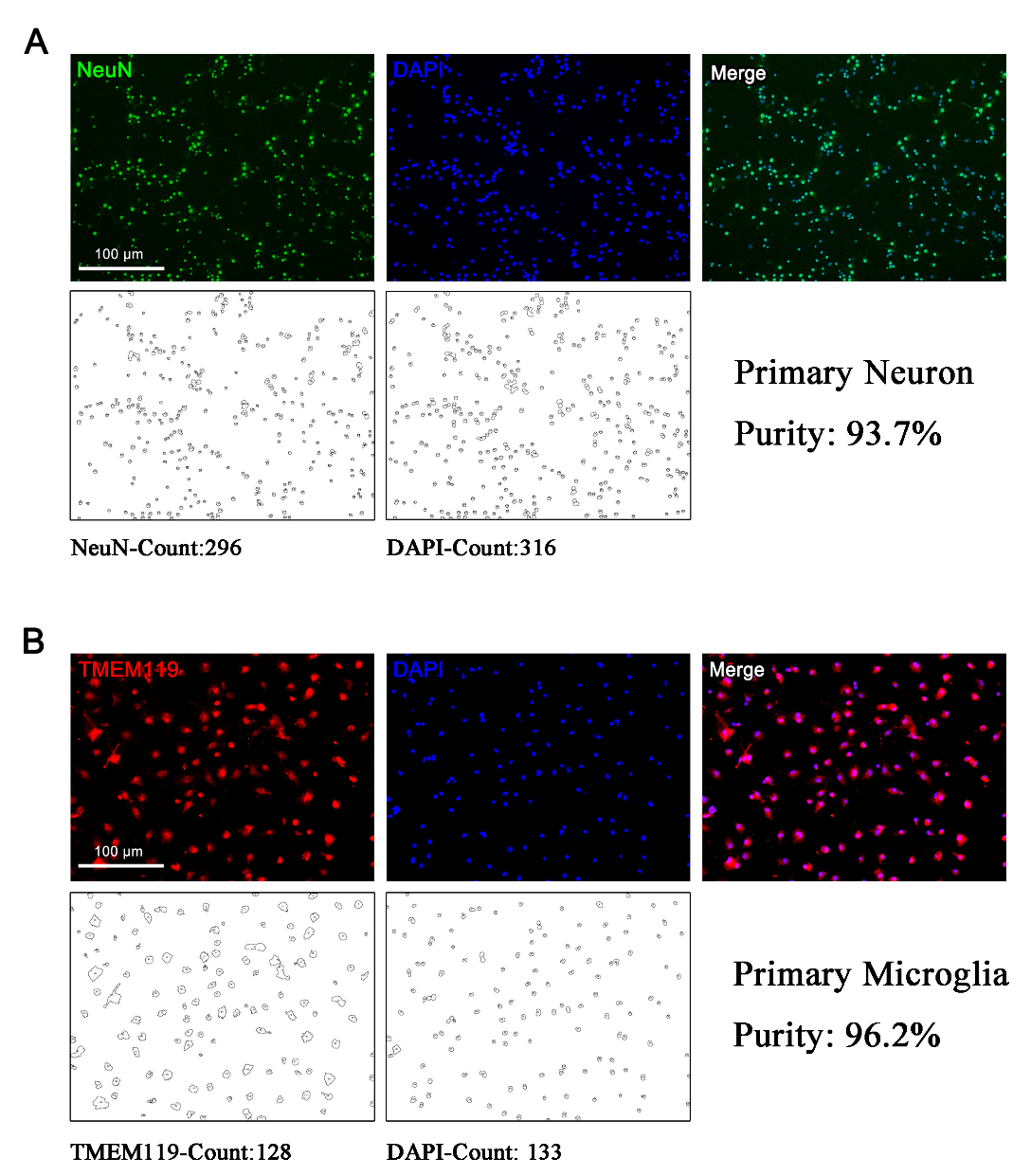


**Additional file 1: Figure S1.** The purity of primary neuron and microglia. **A-B.** Immunofluorescence staining showed the neuron marker NeuN and microglia marker TMEM119 in primary cultured cells. The particle analysis was performed by ImageJ and the purity of primary neuron and microglia was more than 90%.
